# Pleomorphic adenoma of the trachea: A case report

**DOI:** 10.1016/j.ijscr.2023.108499

**Published:** 2023-07-13

**Authors:** Mayu Inomata, Shoei Kuroki, Nobuyuki Oguri, Yuichiro Sato, Fumiya Kawano, Ryo Maeda

**Affiliations:** a Department of Thoracic and Breast Surgery, Faculty of Medicine, University of Miyazaki Hospital, Miyazaki, Japan; b Department of Diagnostic Pathology, Faculty of Medicine, University of Miyazaki Hospital, Miyazaki, Japan

**Keywords:** Case report, Pleomorphic adenoma, Tracheal tumor, Carinal resection, Surgery, Asthma

## Abstract

**Introduction and importance:**

Although pleomorphic adenoma is the most common type of parotid gland tumor, its occurrence in the trachea is rare. Here, we describe a successfully resected pleomorphic adenoma of the trachea in a woman with severe respiratory failure that had been preoperatively misdiagnosed as asthma.

**Case presentation:**

A 69-year-old woman presented to the emergency department with symptoms of worsening dyspnea and subsequent loss of consciousness. She had a history of progressively worsening wheezing and stridor over the course of 2-years and had been diagnosed with asthma. Arterial blood gas sample analysis indicated type II respiratory failure. A chest computed tomographic scan revealed a tumor in the trachea, which was almost completely obstructing the lower tracheal lumen. The tumor was located just above the carina. To alleviate airway constriction and achieve complete resection, carinal resection with reconstruction was performed. The postoperative diagnosis was pleomorphic adenoma of the trachea.

**Clinical discussion:**

Pleomorphic adenoma is a rare tracheal tumor that may present with obstructive airway symptoms that mimic asthma.

**Conclusion:**

Tracheal tumors should be considered in patients with chronic respiratory symptoms that do not improve with medication.

## Highlights

• We describe a successfully resected case of the pleomorphic adenoma of the trachea in a woman with severe respiratory failure.
• Tracheal tumors should be considered when encountering a patient who has chronic respiratory symptoms that do not improve by medication.

## 1 Introduction

Although pleomorphic adenoma is the most common type of tumor of the parotid glands [1], its occurrence in the trachea is relatively rare. The biological behavior and clinical course of pleomorphic adenomas have not been well described owing to the limited number of case reports. In this report, we describe a case of successful resection of a pleomorphic adenoma of the trachea in a woman with severe respiratory failure who was preoperatively misdiagnosed as having asthma. This surgical case was reported in accordance with the SCARE 2020 guidelines [2].

## 2 Case presentation

**Fig. 1:**
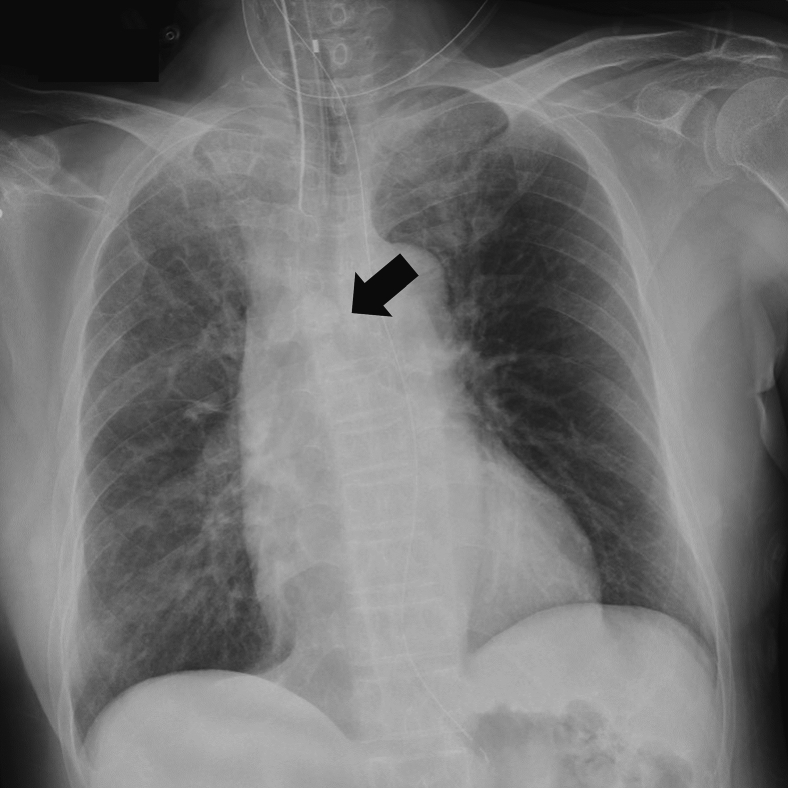
Chest roentgenogram depicting a round mass shadow at the lower tracheal level above the carina (black arrows).

**Fig. 2:**
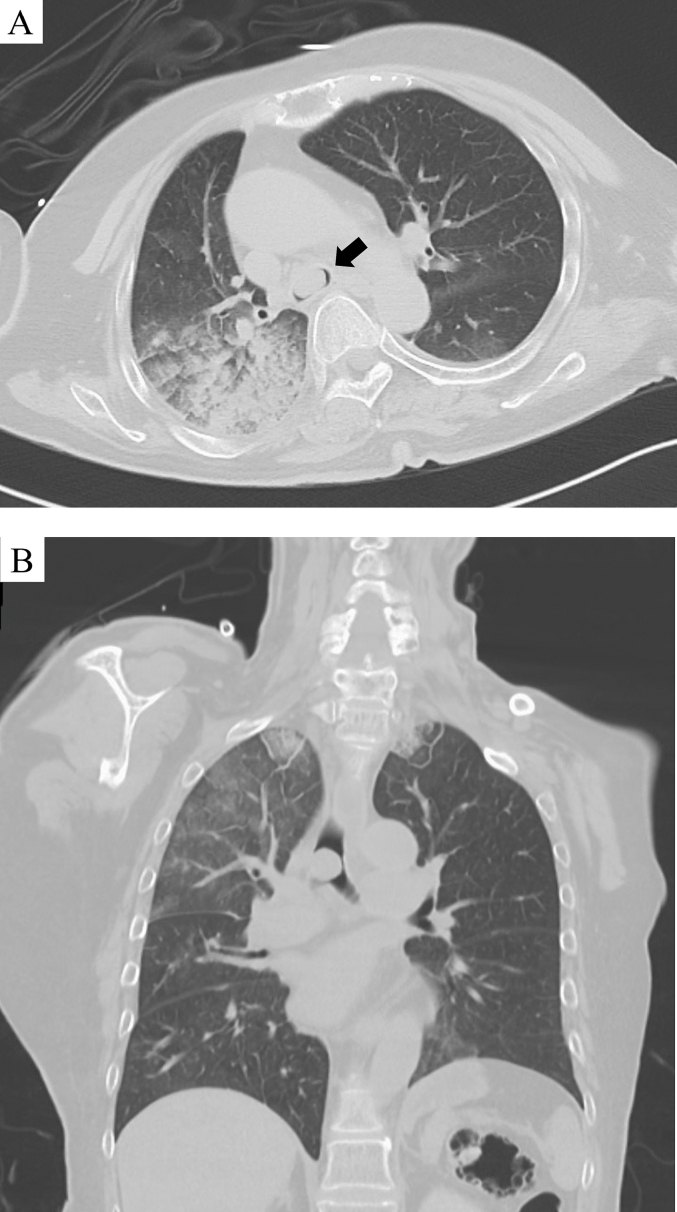
A: Computed tomographic scan of the chest showing a tumor of the trachea and bilateral pneumonia (black arrows); B: Computed tomographic scan with multiplanar reconstruction shows a round lesion obstructing the lumen of the lower trachea located just above the carina.

**Fig. 3:**
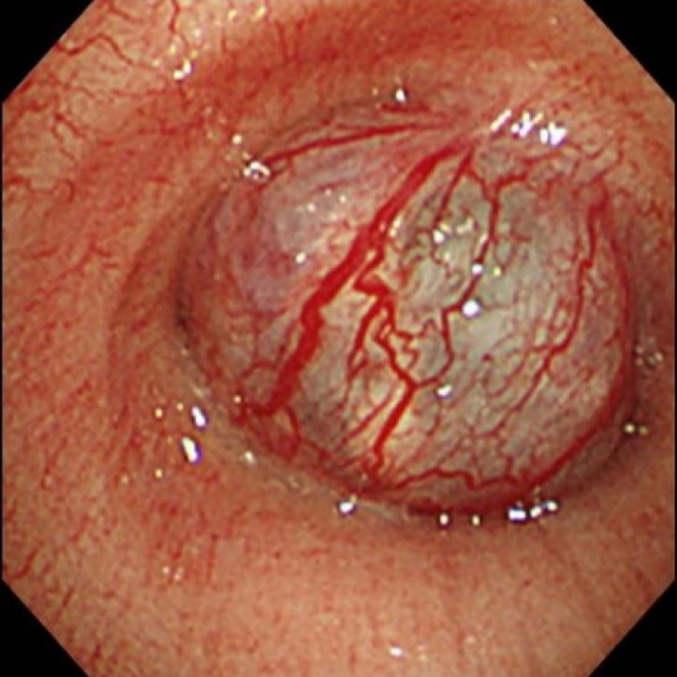
Bronchoscopy shows a vascular neoplasm with a smooth yellow-white colored surface in the lower region of the trachea, with almost complete airway obstruction. (For interpretation of the references to colour in this figure legend, the reader is referred to the web version of this article.)

A 69-year-old woman presented to the emergency department due to worsening dyspnea and subsequent loss of consciousness. She had a 2-year history of progressively worsening wheezing and stridor, and had been diagnosed with asthma. However, treatment with bronchodilators and steroid inhalers failed to resolve the symptoms. An arterial blood gas sample on 15 L/min of oxygen demonstrated type II respiratory failure (pH 6.87, PaO_2_ 63.6 Torr, and PaCO_2_ 181.0 Torr). The patient was immediately intubated with a single-lumen endotracheal tube, but arterial carbon dioxide levels remained elevated (pH 6.81, PaO_2_ 345.0 Torr, and PaCO_2_ 194.0 Torr). Chest roentgenography indicated a round mass shadow at the lower tracheal level above the carina (Fig. 1). An emergent chest computed tomography (CT) scan revealed a tumor in the trachea, which almost completely obstructed the lower tracheal lumen and located just above the carina. Bilateral pneumonia was also observed (Fig. 2A-B). Bronchoscopy revealed a large vascular neoplasm with a smooth yellowish-white surface in the lower region of the trachea, causing almost complete airway obstruction (Fig. 3).

**Fig. 4:**
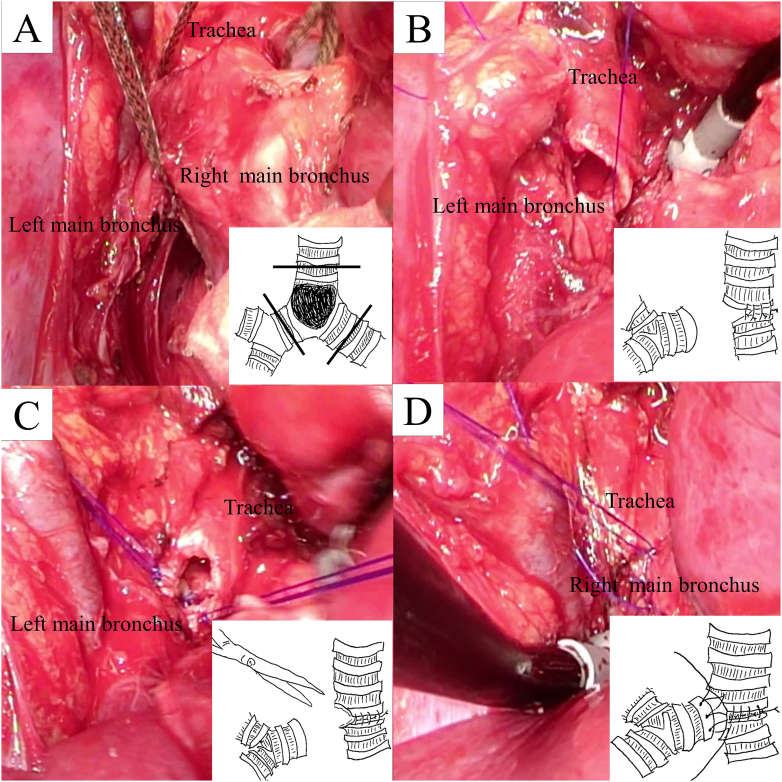
The trachea, left main bronchus, and right main bronchus were transected (A). Following the anastomosis between the trachea and left main bronchus using interrupted sutures, one-third of the primary end-to-end anastomosis remained incomplete (B) and was trimmed to create an appropriate aperture for the secondary end-to-side anastomosis (C). Finally, the right main bronchus was reimplanted into the aperture using running sutures (D).

Because complete excision of the tracheal tumor was considered possible, surgery was performed for complete excision of the tumor to relieve airway constriction. The patient was initially placed in a supine position, and venovenous (VV) extracorporeal membrane oxygenation (ECMO) was performed. The inflow cannula was directed into the right internal jugular vein and the outflow cannula was directed into the right common femoral vein. Ventilation was stopped, and gas exchange was performed via VV ECMO. After positioning the patient in the left decubitus position, a right posterolateral thoracotomy was performed through the fourth intercostal space. We performed carinal resection coupled with reconstruction. Anastomosis was performed as a modified montage-type carinal reconstruction (Fig. 4A-D). A latissimus dorsi flap was used for anastomotic reinforcement. The VV ECMO was dislodged following closure of the thoracotomy, and the patient was extubated for 3 h in the intensive care unit after surgery.

**Fig. 5:**
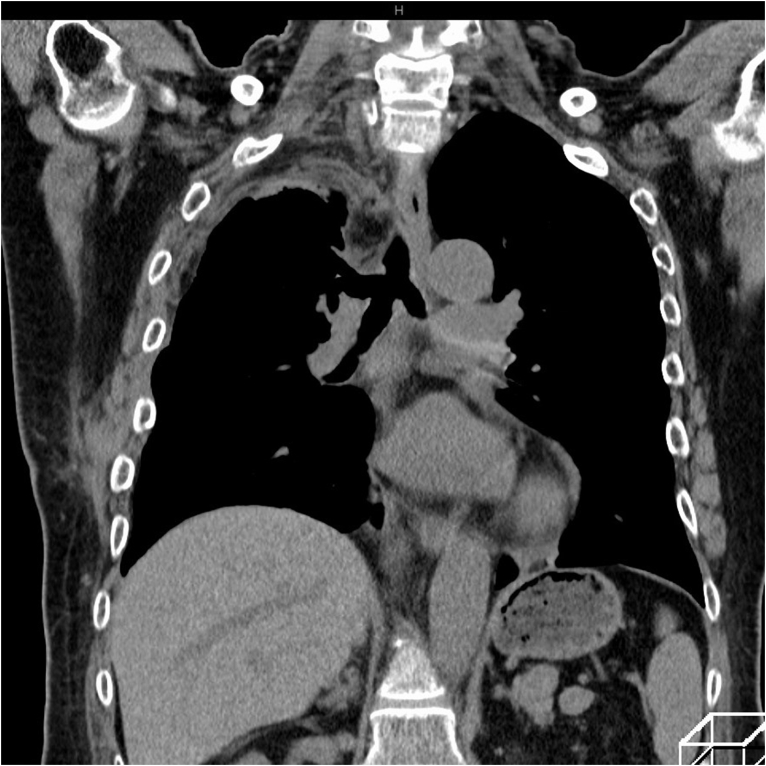
Postoperative computed tomographic scan of the chest performed 2 months postoperatively showed no stenosis at the anastomosed site. A white arrow shows latissimus dorsi flap.

There were no postoperative complications, and the patient was discharged 11 days later. Postoperative CT performed 2 months after surgery demonstrated no evidence of stenosis at the site of the anastomosis (Fig. 5).

**Fig. 6:**
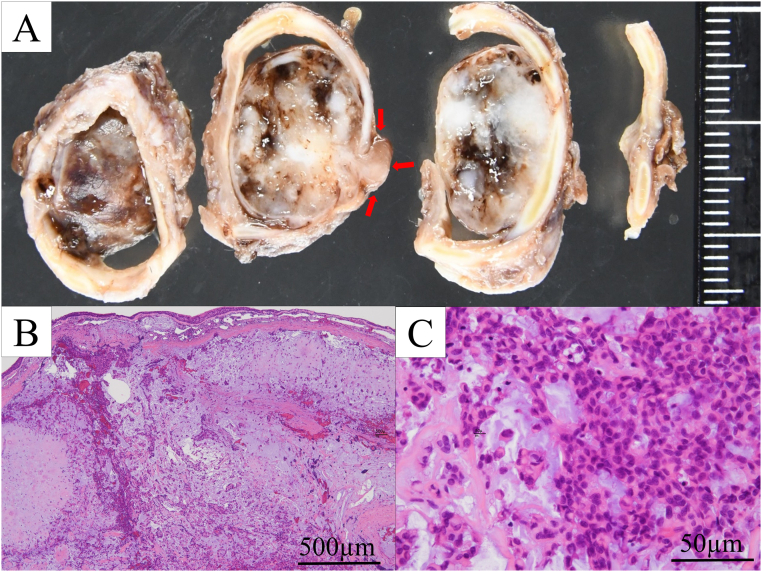
Macroscopic image of the post-fixed specimen. There was a well-demarcated and heterogenous tumor protruding into the luminal side and the surrounding stroma out of the tracheal wall (red arrow) (A). Histologically, biphasic proliferation of chondromyxoid stroma and epithelium component was observed with cartilage formation and hemorrhage. The luminal side of the trachea was covered by non-tumorous respiratory epithelium (H&E, ×20) (B). The epithelial component shows a proliferation of cuboidal to round-shaped cells with oval nuclei and eosinophilic cytoplasm, arranged in nests or singly. Plasmacytoid myoepithelium are also observed. (C). (For interpretation of the references to colour in this figure legend, the reader is referred to the web version of this article.)

Macroscopic findings showed a 15 mm-sized, well-demarcated heterogeneous tumor (Fig. 6A). Histologically, the tumor showed biphasic proliferation of the epithelial component and chondromyxoid stroma with cartilage formation (Fig. 6B-C). Necrosis and mitotic figure were absent. Immunohistochemically, the epithelial tumor cells were diffusely positive for AE1/AE3, CK7, S100, and SMA in various proportions. The Ki-67 labeling index was approximately 1 %. Histological analysis confirmed that the tumor was a pleomorphic adenoma of the trachea, and that the surgical margins were disease-free. She was doing well without local or distant recurrence at her follow-up visit 12 months after surgery, and is currently being followed up as an outpatient.

## 3 Discussion

Primary tumors of the trachea correspond to 0.2 % of all airway neoplasms, affecting 0.1/100,000 individuals per year [3,4]. In adults, 90 % of these lesions are malignant. Squamous cell and adenoid cystic carcinomas are the most common forms of airway neoplasms [5,6]. Benign tumors affecting the airways that are most commonly encountered include granular cell tumors, benign cartilaginous tumors, fibromas, hamartomas, hemangiomas, and squamous papillomas [7]. Pleomorphic adenoma is a common benign neoplasm that affects the salivary glands; however, its occurrence in the trachea is relatively rare [1]. To date, only 40 cases of pleomorphic tracheal adenoma have been reported in the English language literature [[8], [9], [10], [11], [12], [13], [14]]. No clear sex or age predominance was observed. The age of the affected individuals ranged from 8 to 83 years [[8], [9], [10], [11], [12], [13], [14]].

Pleomorphic adenomas are benign neoplasms characterized by mixed epithelial and mesenchymal components [15]. The epithelial elements can exhibit glandular, trabecular, or solid patterns, whereas secretory material is usually found in the ductular structures. The mesenchymal elements, though typically chondroid, can also be myxoid or collagenous, and distinguishing between a pleomorphic adenoma and an adenoid cystic carcinoma can be challenging [1]. Immunohistochemical staining aids in diagnosing pleomorphic adenomas because the results are positive for myoepithelial cell components, cytokeratin, and S100 protein [9]. However, the pathogenesis of pleomorphic adenoma remains unclear. The presence of ectopic tissues in the trachea rather than in the salivary glands or the differentiation of primitive stem cells into ductal structures and chondromyxoid matrices is possible [16,17].

Tracheal tumors have nonspecific clinical manifestations. The most common symptoms of benign tracheal tumors are dyspnea, cough, hemoptysis, wheezing, and stridor [18]. Except for hemoptysis, these clinical presentations can lead to an incorrect diagnosis of asthma. These misleading symptoms often delay the diagnosis of tracheal tumors, and an accurate diagnosis may take several years [19]. Patients rarely notice symptoms until the tracheal tumors grow large enough to obstruct at least 50 % of the tracheal diameter. Our patient, who complained of severe dyspnea with effort, was diagnosed with and treated for asthma. While corticosteroids are an effective treatment for asthma, patients with tracheal tumors, such as our patient, do not typically respond to corticosteroid therapy. This highlights the significance of maintaining a high index of suspicion and utilizing thorough imaging and bronchoscopy procedures to prevent delays in the diagnosis of this condition.

The biological behavior and clinical course of pleomorphic tracheal adenomas have yet to be fully described. Complete excision of the tumor with sufficient margins is the definitive treatment for pleomorphic adenocarcinoma of the salivary gland because inadequate surgical procedures are associated with the risk of recurrence [1]. In addition, pleomorphic tracheal adenomas can become malignant [20]. Surgical resection with sufficient margins is considered the best treatment option for pleomorphic tracheal adenomas. In this case, we successfully performed carinal resection with reconstruction of a tumor involving the tracheal carina. However, carinal resection with reconstruction is one of the most challenging thoracic surgeries, and is associated with high mortality and morbidity [21]. Endobronchial interventions are widely performed for patients with airway stenosis. Liao et al. successfully excised a pleomorphic adenoma of the trachea using cryotherapy and argon plasma coagulation [14]. Endoscopic resection is less traumatic than surgery and allows faster recovery after the procedure. Alternative therapeutic approaches, such as interventional bronchoscopy, may be recommended for palliative purposes in cases where surgical resection cannot be performed owing to the patient's poor condition, advanced age, or refusal to undergo treatment [22].

One case of pleomorphic adenoma of the trachea recurred 10 years after surgical resection [13]. Metastasis to other organs, referred to as “benign metastasizing pleomorphic adenoma” has also been reported [20,23]. Therefore, considering the possible malignant potential of pleomorphic tracheal adenomas, long-term follow-up is mandatory, even after complete surgical resection.

## 4 Conclusion

Pleomorphic adenomas are rare tracheal tumors that may present with obstructive airway symptoms mimicking asthma. Tracheal tumors should be suspected in patients with chronic respiratory symptoms that do not improve with medication. Although complete resection may be the best treatment for pleomorphic adenoma of the trachea, careful follow-up of the postoperative course is important because distant metastasis to other organs has been reported on rare occasions.

## Abbreviations

CT: Computed tomography
VV: Veno-venous
ECMO: extracorporeal membrane oxygenation

## Ethical approval

As it is a case report, ethical approval is exempted by University of Miyazaki Hospital.

## Funding

The authors have no competing interests to declare.

## Author contribution

Dr. Mayu Inomata is the main author and she has designed this report.

Nobuyuki Oguri and Yuichiro Sato have provided us with the histological diagnosis and photos of the slides, and have reviewed.

Shoei Kuroki and Fumiya Kawano have reviewed.

Dr. Ryo Maeda is the writer of this article and corresponding author.

## Guarantor

Dr. Ryo Maeda accepts all responsibility of this article.

## Research registration number

Not applicable.

## Consent

Written informed consent was obtained from the patient for publication of this case report and accompanying images. A copy of the written consent is available for review by the Editor-in-Chief of this journal on request.

## Conflict of interest statement

All author declare that they have no conflicts of interest.
